# Wound-dependent infection by *Nigrospora oryzae* causes sugarcane leaf spot: pathogen characterization and fungicide sensitivity

**DOI:** 10.3389/fpls.2025.1742944

**Published:** 2026-02-10

**Authors:** Ahmad Yusuf Abubakar, Shujie Chen, Qianqi Lu, Sheidu Abdullaziz, Muhammed Mustapha Ibrahim, Hua Zhang, Pinghua Chen

**Affiliations:** 1 Quality Inspection and Testing Center of Sugarcane and Derived Products, Ministry of Agriculture, National Engineering Research Center of Sugarcane, Fujian Agriculture and Forestry University, Fuzhou, China; 2 Key Laboratory of Ministry of Education for Genetics, Breeding and Multiple Utilization of Crops, College of Agriculture, Fujian Agriculture and Forestry University, Fuzhou, China; 3 Fujian Agriculture and Forestry University, Fuzhou, China

**Keywords:** bioenergy production, crop cultivation, fungal pathogen, fungicide sensitivity, *Nigrospora oryzae*, sugarcane, wound infection

## Abstract

**Introduction:**

*Nigrospora oryzae* is increasingly detected in sugarcane fields, but its infection biology and effective chemical control options remain unclear.

**Methods:**

We isolated the causal fungus from symptomatic sugarcane leaves and identified it using morphology and ITS phylogeny. Pathogenicity was tested on cultivar ROC22 using wounded and unwounded inoculations (mycelial plugs and spore suspensions). We assessed fungal growth across temperatures and pH, quantified host chlorophyll and defense-related responses (POD, SOD, PAL, and MDA), and evaluated sensitivity to commonly used fungicides using growth-inhibition assays.

**Results:**

The isolate (MF1) clustered with *N. oryzae* and caused lesions only on mechanically wounded leaves. Infection reduced chlorophyll content and increased POD, SOD, PAL activities and MDA accumulation. Optimal growth occurred around 25–30°C and near neutral pH. Among tested fungicides, pyraclostrobin + metiram and difenoconazole showed the strongest inhibition (lowest EC_50_), whereas several others were weak or ineffective.

**Discussion:**

These results indicate *N. oryzae* acts as a wound-dependent opportunistic pathogen of sugarcane. The sensitivity profile provides baseline guidance for integrated management and resistance-aware fungicide selection.

## 1 Introduction

Sugarcane (*Saccharum officinarum* L.) is a key crop for sugar and bioenergy production worldwide, but its productivity is increasingly constrained by emerging foliar diseases driven by intensive cultivation and changing environmental conditions (Ali et al., 2021; Pang et al., 2021). Advances in molecular tools and genomics have improved pathogen detection and our understanding of host responses, yet many newly reported pathogens remain poorly characterized in sugarcane (Li et al., 2023; Ramesh Sundar et al., 2015). *Nigrospora oryzae* is an opportunistic fungal species with a broad host range, including ginger, wild rice, cotton, yam, and tobacco, and causes leaf spotting and blight symptoms (Liu et al., 2021; Han et al., 2021; Lu et al., 2023; Wang et al., 2022; Liu et al., 2022). Reports across hosts have described similar symptomology (circular to irregular necrotic lesions often surrounded by chlorotic halos), yet the pathogenic mechanisms, infection requirements, and impacts on host physiology vary among hosts and remain incompletely defined (Qiu et al., 2022; Widmer et al., 2006). Although *N. oryzae* has been detected on sugarcane using morphological and internal transcribed spacer (ITS) sequence data (Chen, 2020), experimental confirmation of pathogenicity on sugarcane and characterization of the infection biology are lacking. In particular, it is unclear whether *N. oryzae* can directly infect intact sugarcane tissue or acts primarily as a wound-dependent opportunist, information that is critical for framing management interventions given the prevalence of mechanical injury from harvesting, insects, and weather events. Equally important is knowledge of the host’s physiological response to infection. Changes in chlorophyll content, lipid peroxidation [malondialdehyde (MDA)], and the activities of antioxidant and defense enzymes [superoxide dismutase (SOD), peroxidase (POD), and phenylalanine ammonia-lyase (PAL)] are common indicators of pathogen-induced stress and can inform both diagnosis and breeding for tolerance (Geetha et al., 2005; Song et al., 2022; Wang et al., 2024). Finally, chemical control remains a frontline option for growers, but the effectiveness of commonly used fungicides against *N. oryzae* in sugarcane and the associated resistance risks have not been systematically evaluated (Massi et al., 2021; Yin et al., 2023; Hawkins, 2024).

To fill these gaps, the present study 1) isolated and identified the causal fungus from symptomatic sugarcane leaves using morphology and ITS phylogeny, 2) tested pathogenicity and evaluated whether infection is wound-dependent, 3) characterized host physiological and biochemical responses following inoculation, and 4) screened commonly used fungicides for *in vitro* and *in planta* efficacy. By integrating pathogen identification, infection biology, host response metrics, and fungicide sensitivity data, this work provides a comprehensive assessment of *N. oryzae* as a sugarcane foliar pathogen. It delivers baseline information for diagnosis and integrated management.

## 2 Materials and methods

### 2.1 Plant materials

Seedlings of sugarcane cultivar ROC22 were grown in a controlled greenhouse at 25°C ± 2°C, 60%–70% relative humidity (RH), and a 12-hour light/12-hour dark photoperiod (≈200 μmol m^−2^ s^−1^). These same conditions were used for collecting diseased material and for all downstream experiments to ensure comparability. Healthy ROC22 plants maintained under identical conditions served as inoculation recipients and mock controls.

### 2.2 Pathogen isolation, identification, and microscopy

#### 2.2.1 Isolation

Symptomatic sugarcane leaves showing typical leaf-spot lesions were rinsed under running tap water, surface-sterilized in 70% ethanol for 30 s and 1%–2% sodium hypochlorite for 60–90 s, then rinsed three times in sterile distilled water, and blotted dry. Small tissue pieces (≈5 × 5 mm) taken from the lesion margins were placed onto potato dextrose agar (PDA) amended with streptomycin (50 μg mL^−1^) to suppress bacteria and incubated at 28°C in the dark. Emerging colonies with uniform morphology were subcultured until pure cultures were obtained.

#### 2.2.2 Culture

Pure cultures were obtained by hyphal-tip (or single-spore) isolation and maintained on PDA (200 g potato, 20 g dextrose, and 15 g agar, to 1 L) at 25°C–28°C. For downstream assays, 7-day-old colonies grown on PDA were used to prepare inoculum by flooding plates with sterile distilled water containing 0.01% Tween-20, gently scraping to dislodge conidia, filtering through sterile gauze, and adjusting the suspension to 1 × 10^6^ conidia mL^−1^ with a hemocytometer; where mycelial inoculation was required, 5-mm agar plugs were cut from the actively growing margin.

#### 2.2.3 Inoculum preparation

The purified *N. oryzae* isolate was cultured on PDA (25°C, 5–7 days). Conidia were harvested in sterile water containing 0.01% Tween-20, filtered through sterile gauze, and adjusted to ~1 × 10^6^ conidia mL^−1^ using a hemocytometer. Mock inoculum was a carrier only.

#### 2.2.4 Inoculation methods

**Figure 1:**
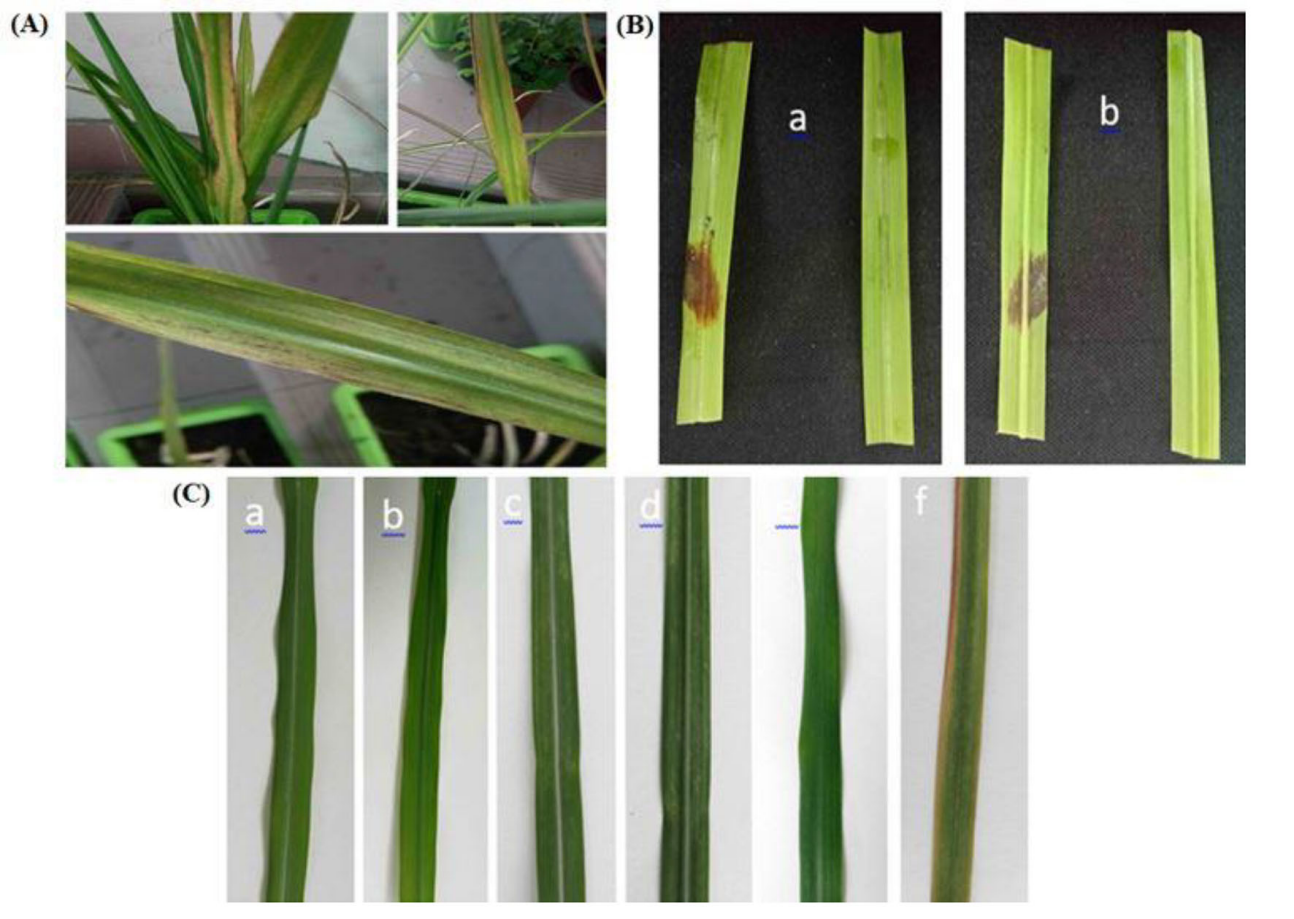
Symptom development on sugarcane (ROC22) leaves following inoculation with *Nigrospora oryzae*. **(A)** Leaf symptoms of the disease in the field. **(B)** Wound-drop inoculation (needle-prick + 10 μL conidial suspension, ~1 × 10^6^ mL^−1^) at 72 hpi on the adaxial surface showing reddish-brown necrotic spots confined to wounded sites. **(C)** Progression at 5–6 dpi, with lesion coalescence and early gray sporulation visible on the abaxial surface. pi, post inoculation.

To compare infection routes, three inoculation methods illustrated in **Figure 1** were first screened on healthy ROC22 leaves: 1) wound-drop: two to three needle pricks per site, and then 10 μL conidial suspension was applied; 2) spray: fine mist application to the runoff of the same suspension; and 3) non-wound droplet: 10 μL droplet placed on intact leaf surface.

After inoculation, plants were kept at high humidity (under a plastic cover for ~24 hours) and then returned to greenhouse conditions.

#### 2.2.5 Morphology (microscopic examination)

Colony characteristics (color, texture, margin, and growth rate) were recorded on PDA after 7 days. Microscopic features were examined from slide mounts prepared in lactophenol cotton blue. Conidiogenous structures and conidia were imaged using a compound microscope (Olympus BX53; Olympus Corporation, Tokyo, Japan) at ×400 to ×1,000. At least 30 conidia were measured to estimate size (mean ± SD). Morphological characteristics were compared with descriptions of *Nigrospora* spp.

### 2.3 DNA extraction/ITS

Genomic DNA was extracted from fresh mycelium (≈3–5 days on PDA) using the cetyltrimethylammonium bromide (CTAB) (Doyle and Doyle, 1987) method. The ITS region was amplified with primers ITS1 (5′-TCCGTAGGTGAACCTGCGG-3′) and ITS4 (5′-TCCTCCGCTTATTGATATGC-3′) (White et al., 1990). PCR conditions were as follows: 95°C for 3 min; 35 cycles of 95°C for 30 s, 55°C for 30 s, and 72°C for 60 s; final extension at 72°C for 5 min. Amplicons were verified on a 1%–1.5% agarose gel and Sanger-sequenced bidirectionally.

### 2.4 BLAST and phylogenetic analysis

Consensus ITS sequences were queried against the NCBI nr/nt databases using BLAST to confirm identity (Altschul, 1997). BLASTn of the ITS sequence showed ≥99% identity to *N. oryzae* (reference GenBank accession KU254608.1). Sequences from this study will be deposited in GenBank upon acceptance. Sequences were aligned with MAFFT v7 (Katoh and Standley, 2013). ModelFinder in IQ-TREE selected the best-fit substitution model (Kalyaanamoorthy et al., 2017). Maximum-likelihood trees were inferred with IQ-TREE 2 using 1,000 ultrafast bootstrap replicates (Minh et al., 2020). Trees were rooted with *Arthrinium arundinis* (Apiosporaceae), and node support ≥95 was considered strong.

### 2.5 Pathogenicity assays (Koch’s postulates)

Mycelial plugs and spore suspensions were used to assess pathogenicity in both *in vitro* (detached leaves) and *in vivo* (attached leaves) settings. Just before inoculation, leaves were lightly punctured with a sterile needle for wounding treatments; 5-mm PDA plugs with growing mycelium were applied to the leaf surface in mycelial-plug assays; sterile PDA plugs were used as controls. The inoculum was evenly applied to the specified spot in spore-suspension experiments, while controls were given sterile water. Symptoms were noted at 12, 24, 36, 48, 60, and 72 hours after inoculation, and the plants were maintained in a climate chamber (PRX-450A, Ningbo Saifu Laboratory Equipment, Ningbo, Zhejiang, China) under carefully monitored conditions.

### 2.6 Mycelial growth assays (temperature and pH)

#### 2.6.1 Temperature

Four-millimeter-diameter agar plugs taken from the actively growing margins of 7-day-old colonies were placed at the center of fresh PDA plates, sealed, and incubated in the dark for 5 days. Colony diameter was measured along two perpendicular axes, with three replicates per treatment. Mycelial growth of *N. oryzae* on PDA was evaluated at 15°C, 20°C, 25°C, 30°C, and 35°C. The effect of temperature on colony diameter was significant [one-way analysis of variance (ANOVA), *p* < 0.001].

#### 2.6.2 pH

PDA was adjusted to nine pH levels (4, 5, 6, 7, 8, 9, 10, 11, and 12) using 0.1 M L^−1^ HCl to evaluate the impact of pH on *N. oryzae* growth. Four-millimeter-diameter agar plugs taken from the actively growing margins of 7-day-old colonies were inoculated onto PDA plates at the designated pH. Plates were sealed and incubated at 28°C in the dark for 5 days. Colony diameter was then measured along two perpendicular axes. Each pH treatment was replicated three times, and pH had a significant effect on colony diameter (one-way ANOVA, *p* < 0.001).

### 2.7 Physiological and biochemical assessments

Leaf tissues were taken every 24 hours for 6 days post inoculation (0–6 dpi) to measure MDA, a marker of lipid peroxidation, and to assess the activity of SOD, POD, and PAL. Three biological replicates were used for each treatment and time point in all experiments; technical replicates were averaged before statistical analysis. Commercial colorimetric kits (Nanjing Jiancheng Bioengineering Institute, Nanjing, China; cat. nos.: SOD, A001-3-1; POD, A084-3-1; PAL, A137-1-1; and MDA, A003-1-2) were used to test SOD, POD, PAL, and MDA according to the manufacturer’s instructions. MDA is reported as nmol g^−1^ fresh weight (FW), while enzyme activity is expressed as U mg^−1^ protein. Total chlorophyll (*a* + *b*) was measured spectrophotometrically at 663 and 645 nm from 80% (v/v) acetone extracts using a UV–Vis spectrophotometer (Shimadzu UV-1800, Kyoto, Japan) with 1-cm quartz cuvettes; concentrations were calculated per Lichtenthaler (1987) and expressed as mg g^−1^ FW. Enzyme activity data were analyzed using a two-way ANOVA with treatment and time (0–6 dpi) as fixed factors and their interaction, implemented in R using the drc package (Ritz et al., 2015).

### 2.8 Fungicide screening (*in vitro* and *in planta*)

Eight fungicides commonly used in sugarcane—chlorothalonil, difenoconazole (10%), copper hydroxide, pyraclostrobin + metiram (60%), carbendazim, metalaxyl + hymexazol, myclobutanil, and zhongshengmycin—were evaluated against *N. oryzae*. For the *in vitro* assay, PDA was amended with each fungicide at 50, 25, 12.5, and 6.25 μg mL^−1^. Plates were inoculated with *N. oryzae* and incubated. Mycelial growth inhibition by fungicides was assessed using colony area rather than diameter to obtain more accurate estimates of radial expansion. Colony radius (r) was measured in two perpendicular directions, and colony area (A) was calculated as A = πr^2^. Colony growth was quantified from colony area and computed from measured diameter: A = π(D/2)^2^. For each fungicide, the inhibition rate (%) was calculated relative to untreated controls, as follows:


$$\frac{Acontrol-\;\text{Atreatment}}{\text{Acontrol}}\;\times \;100$$

Dose–response data were fitted using a log-dose probit regression model to obtain toxicity regression equations, correlation coefficients, and EC_50_ values. Regression curves with 95% confidence intervals were generated to visualize fungitoxicity trends.

For the plant assay, detached-leaf or intact-plant tests were conducted under the same greenhouse conditions using a formulation of 60% pyraclostrobin + metiram and 10% difenoconazole and applied at labeled rates. Disease severity or mycelial growth inhibition was recorded, and treatments were compared statistically at α = 0.05.

### 2.9 Statistical analysis

Data were analyzed using SPSS v18.0, and figures were prepared using GraphPad Prism v8.0. Differences among treatments were assessed using one-way ANOVA. When significant differences were detected, means were separated using Tukey’s test at *p* < 0.05. In figures, values are presented as means ± standard error (SE), and groups that differ statistically are indicated by different letters. Dose–response data were fitted with a four-parameter log-logistic (LL.4) model using the drc package in R (Ritz et al., 2015) to estimate EC_50_ and 95% confidence intervals.

## 3 Results

### 3.1 Isolation and morphological characteristics of the pathogen

**Figure 2:**
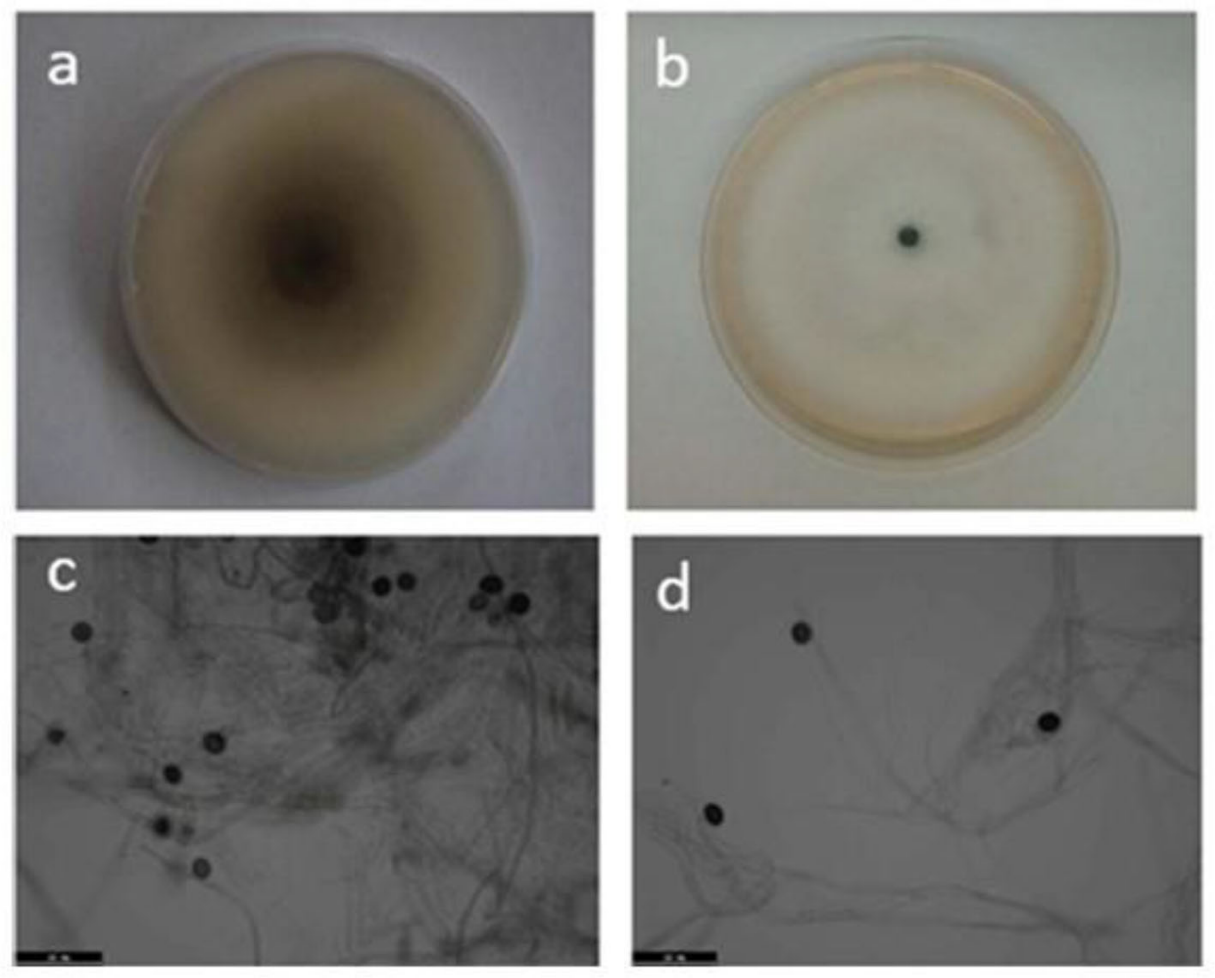
Morphological characteristics of *Nigrospora oryzae* on PDA. **(a)** Reverse side of colony after 7 days. **(b)** Surface view of colony. **(c)** Conidia (scale bar = 50 μm). **(d)** Spores and hyphae (scale bar = 50 μm). PDA, potato dextrose agar.

A fungal isolate was consistently recovered from symptomatic sugarcane leaves (**Figures 1A–C**). Colonies on PDA were initially white and later darkened to gray-black with dense aerial mycelia (**Figures 2A, B**). Conidia were black, spherical to slightly ellipsoid, and measured approximately 12–15 μm in diameter (**Figures 2C, D**). These characteristics are consistent with *Nigrospora* species.

### 3.2 Molecular identification of the pathogen

**Figure 3:**
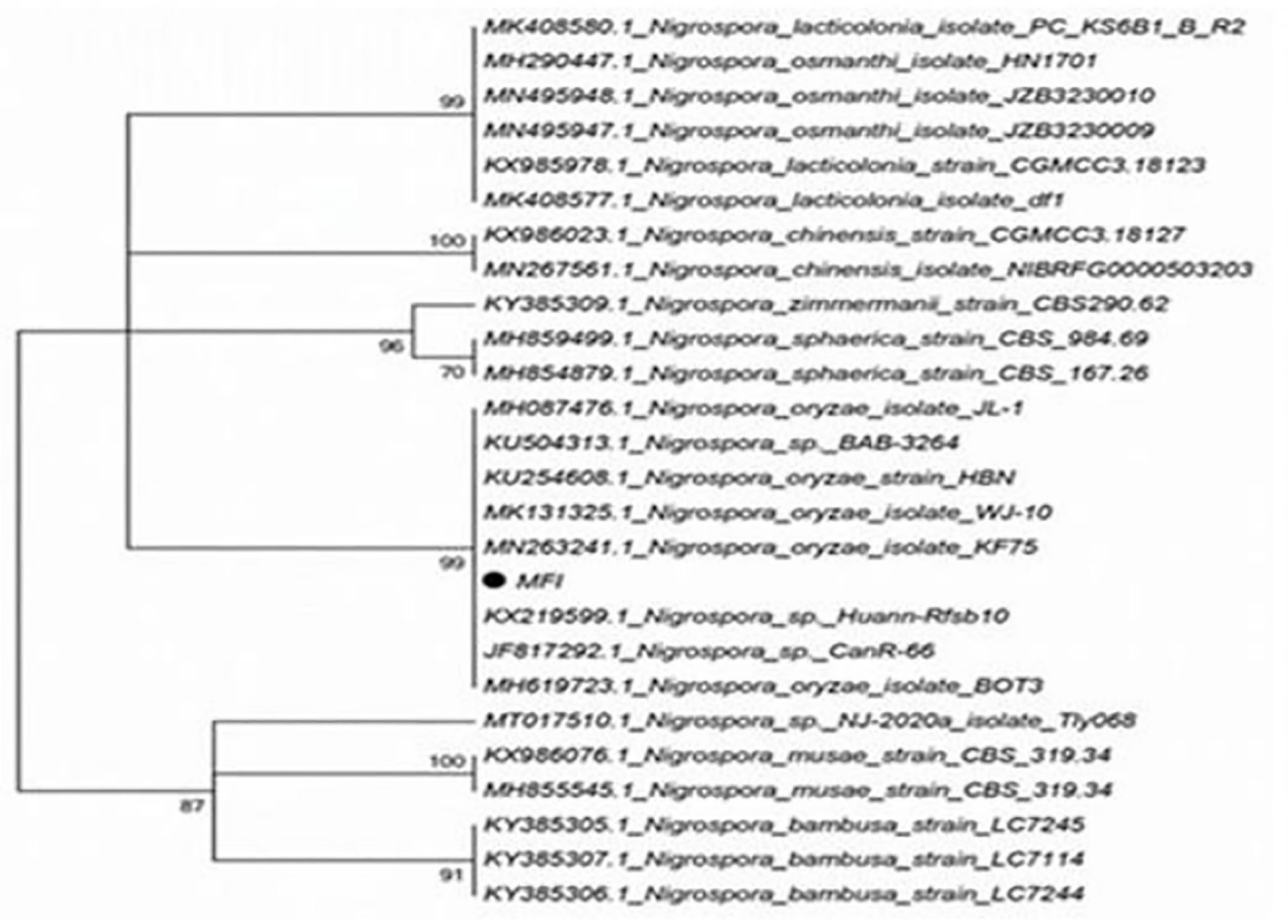
Phylogenetic tree based on ITS sequences. The neighbor-joining (NJ) tree was constructed in MEGA v5.0 using ITS sequences from the isolate and reference strains. Bootstrap values (1,000 replicates) are shown at branch nodes. The sugarcane isolate (MF1) is highlighted in bold and clusters with *Nigrospora oryzae* reference strains (reference sequence GenBank accession no. KU254608.1). ITS, internal transcribed spacer.

Gel electrophoresis of the PCR-amplified ITS product revealed a specific fragment of approximately 500 bp, confirming successful amplification. The purified PCR product was sequenced, and the resulting sequence was analyzed using BLAST on the NCBI website (www.ncbi.nlm.nih.gov). Our ITS sequence matched *N. oryzae* with ≥99% identity (reference: KU254608.1). In accordance with the morphological observations, the isolate clustered within the *N. oryzae* clade in a phylogenetic tree constructed in MEGA v5.0 using the neighbor-joining method with 1,000 bootstrap replicates (**Figure 3**). Based on these combined results, the pathogen was identified as *N. oryzae*. The associated disease in sugarcane was named as *Nigrospora* leaf spot, following nomenclature guidelines for plant fungal diseases (Zheng, 2012).

### 3.3 Pathogenicity assays and wound defense

**Figure 4:**
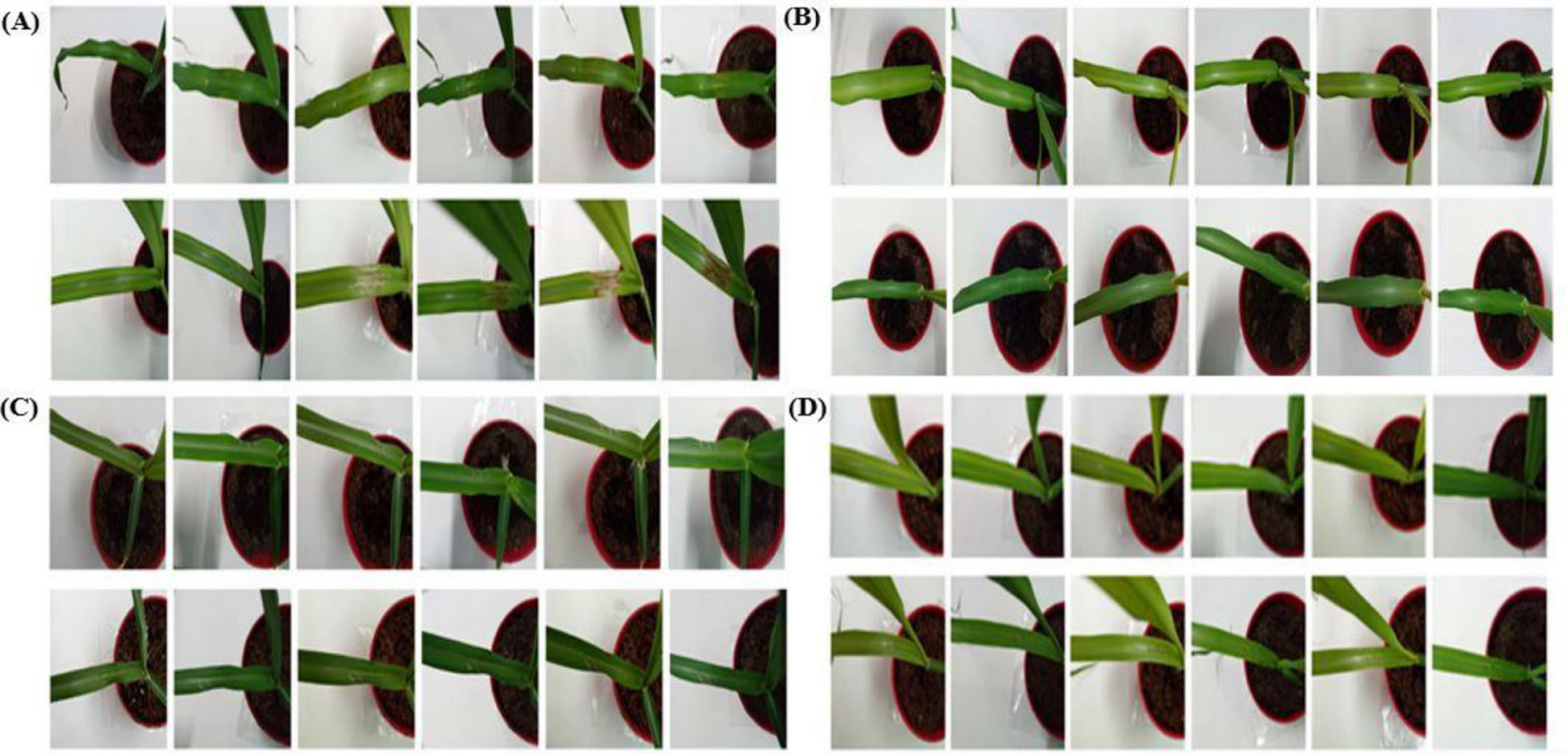
Pathogenicity assays of *Nigrospora oryzae* on sugarcane leaves using different inoculation methods. **(A)** In vivo inoculation with mycelial plugs on wounded leaves: (a) control leaves treated with sterile PDA and (b) leaves inoculated with fungal plugs. **(B)** In vivo inoculation with mycelial plugs on unwounded leaves: (a) control leaves treated with sterile PDA and (b) inoculated leaves. **(C)** In vivo inoculation with spore suspension on wounded leaves: (a) control leaves treated with sterile water and (b) inoculated leaves. **(D)** In vivo inoculation with spore suspension on unwounded leaves: (a) control leaves treated with sterile water and (b) inoculated leaves. All panels show progression of symptoms at 12, 24, 36, 48, 60, and 72 hours post inoculation (left to right). PDA, potato dextrose agar.

Necrotic lesions developed consistently only on mechanically wounded leaves inoculated with either spore suspensions or mycelial plugs of *N. oryzae*. No symptoms were observed on unwounded inoculated leaves or on any of the control treatments during the 72-hour observation period. Wounded leaves inoculated with the pathogen exhibited visible necrotic lesions within 3–4 days (**Figures 4A–C**), whereas unwounded leaves remained symptomless (**Figures 4B, D**). The pathogen was successfully re-isolated from symptomatic tissue, thereby fulfilling Koch’s postulates and confirming its pathogenicity.

### 3.4 Pathogenicity and effects on chlorophyll content

Mechanical wounding was essential for successful infection by *N. oryzae*. Wounded leaves inoculated with mycelial plugs developed necrotic lesions within 24 hours, which enlarged progressively over the next 72 hours (**Figure 4A**). Detached leaves inoculated with mycelial plugs exhibited even faster symptom onset (24–36 hours), indicating greater susceptibility of excised tissue compared with intact plants (**Figure 4B**). Inoculation with spore suspensions produced lesions only on wounded leaves, with visible necrosis appearing at 48–60 hours and becoming more pronounced by 72 hours (**Figure 4C**). Throughout the 72-hour observation period, unwounded leaves inoculated with spore suspensions remained symptomless (**Figure 4D**). The pathogen was successfully re-isolated from symptomatic tissues, completing Koch’s postulates. Infection significantly reduced chlorophyll *a*, chlorophyll *b*, and total chlorophyll in wounded inoculated leaves compared with controls (**Table 1**). No significant changes were observed in unwounded or mock-inoculated leaves.

**Table 1:** Radial mycelial growth of *Nigrospora oryzae* on PDA after 5 days at 28°C in the dark following exposure to fungicides.

Fungicide	Concentration (μg/mL)	Diameter (mm)	Area (mm^2^)	Area-based inhibition (%)
75% Chlorothalonil	50	30.3	720.75	86.98
	25	37.08	1,080.36	80.03
	12.5	47.09	1,742.44	69.59
	6.25	51.3	2,066.92	64.42
	0	60.6	2,885.44	—
10% Difenoconazole	50	13.5	143.14	94.68
	25	18.1	257.26	92.23
	12.5	24.8	483.92	87.56
	6.25	41	1,320.25	66.13
	0	59.3	2,762.76	—
46% Copper hydroxide	50	57.4	2,589.02	5.8
	25	57.5	2,597.73	5.22
	12.5	58.3	2,671.82	2.68
	6.25	59.8	2,810.26	0.58
	0	60.3	2,858.84	—
60% Pyraclostrobin + metiram	50	6.7	35.23	99.4
	25	8	50.27	99.16
	12.5	8.2	52.78	99.05
	6.25	8.4	55.38	98.98
	0	60.1	2,838.17	—
50% Carbendazim	50	52.3	2,147.91	24.84
	25	58.3	2,671.82	0.9
	12.5	59.7	2,802.64	0.03
	6.25	59.8	2,810.26	0
	0	60.3	2,858.84	—
3% Metalaxyl + hymexazol	50	4.5	15.9	97.66
	25	52.3	2,147.91	23.6
	12.5	54.7	2,350.76	17.55
	6.25	52.3	2,147.91	23.6
	0	59.8	2,810.26	—
25% Myclobutanil	50	37.9	1,127.96	71.42
	25	39.9	1,250.91	67.33
	12.5	41.7	1,364.75	65.11
	6.25	42.6	1,425.48	63.69
	0	59.9	2,823.47	—
3% Zhongshengmycin	50	53.6	2,256.54	20.25
	25	54.5	2,332.9	16.22
	12.5	57.9	2,633.94	5.91
	6.25	60	2,827.43	0.62
	0	60.1	2,838.17	—

Inoculum was a 4-mm agar plug taken from a 7-day culture (n = 3 plates per treatment). Values are means ± SD of colony diameter (mm). Percent inhibition was calculated as [(D_control_ − D_treatment_)/D_control_] ×100. Within each concentration, different letters indicate significant differences among fungicides (*post-hoc* multiple comparison at α = 0.05).

Formulation abbreviations: WP, wettable powder; WG, water-dispersible granule; EC, emulsifiable concentrate; PDA, potato dextrose agar.

### 3.5 *In vitro* pathogenicity assays using mycelial plugs and spore suspensions

**Figure 5:**
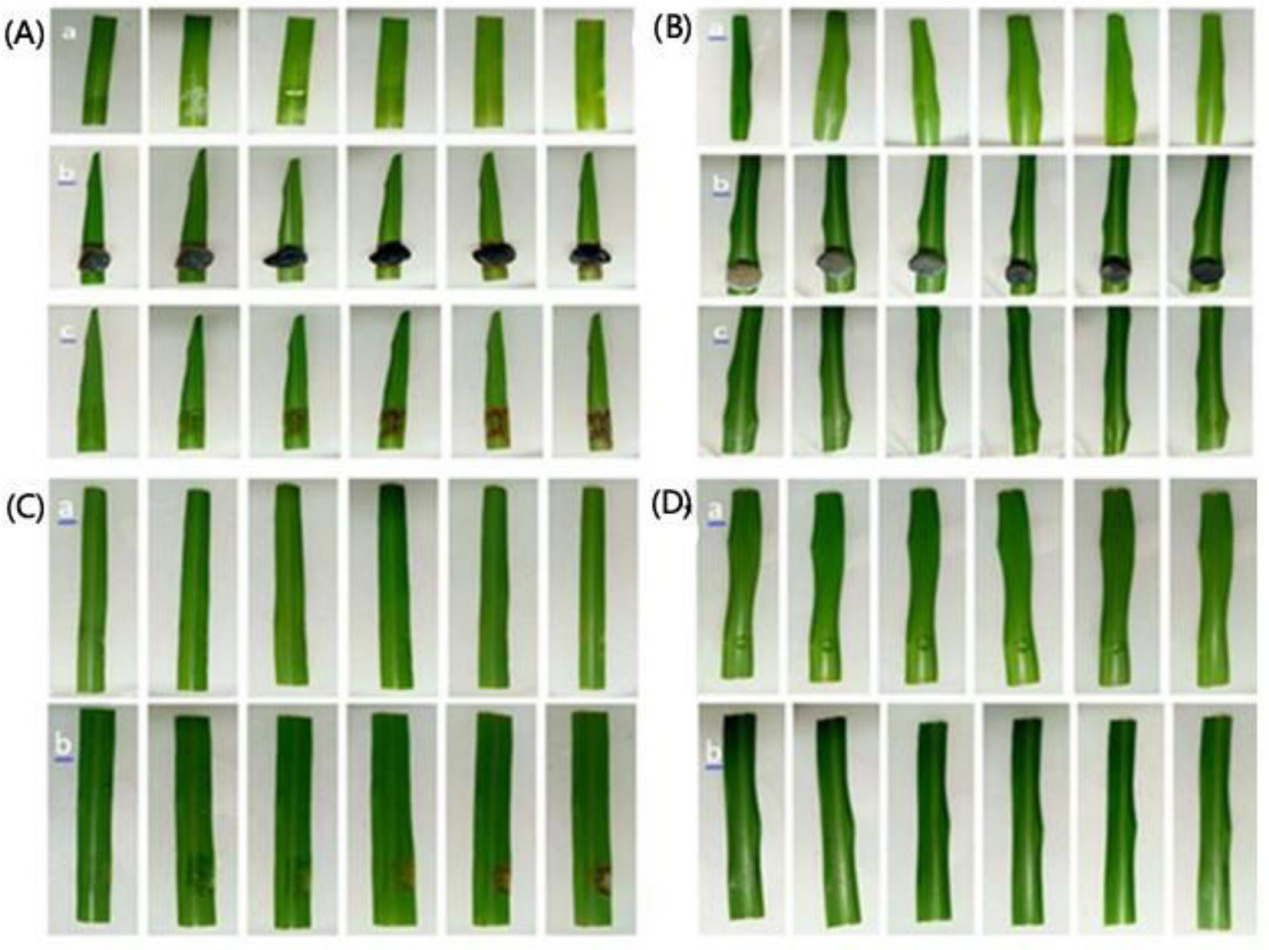
Pathogenicity assays of *Nigrospora oryzae* on sugarcane leaves under different conditions. **(A)** In vitro inoculation with mycelial plugs on wounded leaves: (a) control leaves treated with sterile PDA, (b) leaves being inoculated, and (c) inoculated leaves. **(B)** In vivo inoculation with mycelial plugs on unwounded leaves: (a) control leaves treated with sterile PDA and (b) leaves being inoculated and (c) inoculated leaves. **(C)** In vitro inoculation with spore suspension on wounded leaves: (a) control leaves treated with sterile water and (b) inoculated leaves. **(D)** In vitro inoculation with spore suspension on unwounded leaves: (a) control leaves treated with sterile water and (b) inoculated leaves. All panels show symptom progression at 12, 24, 36, 48, 60, and 72 h post inoculation (left to right). PDA, potato dextrose agar.

*In vitro* pathogenicity tests further demonstrated that *N. oryzae* rapidly initiates lesion formation on wounded sugarcane leaves. When mycelial plugs were applied to injured tissue, faint reddish lesions developed within 24–36 hours and expanded progressively, with visible mycelial proliferation by 72 hours (**Figure 5A**). Detached leaves inoculated with mycelial plugs showed similar symptoms, including red patches and central yellowing by 48 hours (**Figure 5B**). Spore suspension inoculation also resulted in disease development, but with a slightly slower onset. Mild reddening at wound sites appeared at approximately 36 hours, followed by the enlargement of lesions with reddish-brown margins and mycelial development by 72 hours (**Figure 5C**). Throughout the 72-hour observation period, unwounded leaves inoculated with spore suspensions remained symptomless, resembling the uninoculated controls (**Figure 5D**). All control treatments (including sterile PDA plugs and sterile water) failed to produce lesions, suggesting that symptom development was pathogen-specific.

### 3.6 Effects of temperature and pH on mycelial growth

**Figure 6:**
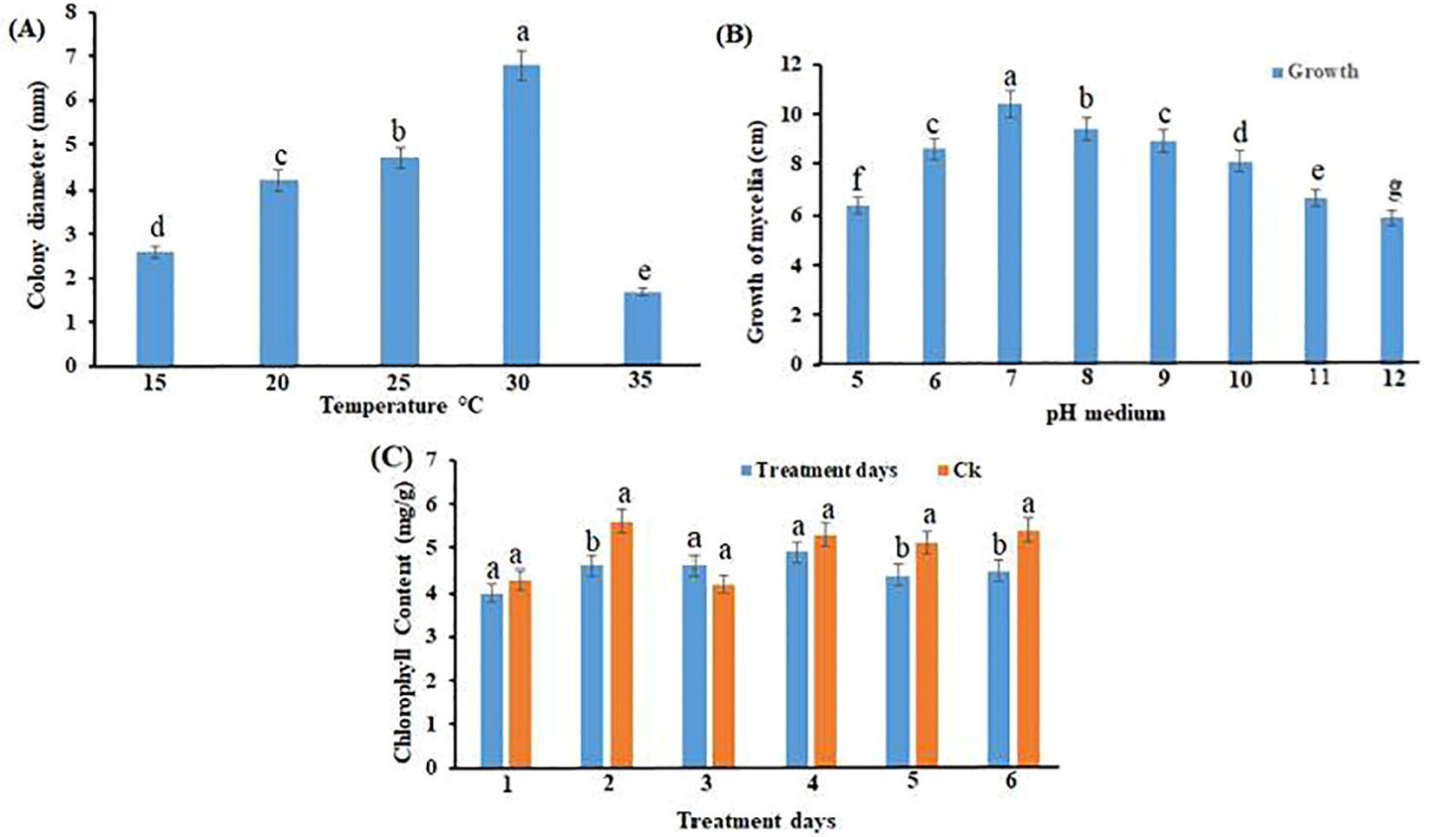
Growth of *Nigrospora oryzae* under different environmental conditions. **(A)** Effect of temperature on mycelial growth. **(B)** Effect of pH on mycelial growth. **(C)** Effect of *N. oryzae* infection on chlorophyll content of sugarcane seedlings. Values are means ± SD (n = 3). Different letters above error bars indicate significant differences (ANOVA, *p* < 0.05). Ck, control.

Temperature significantly influenced radial mycelial growth on PDA (**Figure 6A**). The isolate exhibited maximal colony expansion at 25°C–30°C. Growth declined markedly at 20°C and 35°C, and no growth occurred at 40°C. Differences among temperatures were statistically significant (*p* < 0.05). Similarly, pH strongly affected mycelial development (**Figure 6B**). The fastest growth was recorded at pH 7, with moderate growth at pH 5–6 and pH 8. Mycelial growth was substantially reduced under more acidic (pH 4) or alkaline (pH 9) conditions.

### 3.7 Physiological and biochemical changes

**Figure 7:**
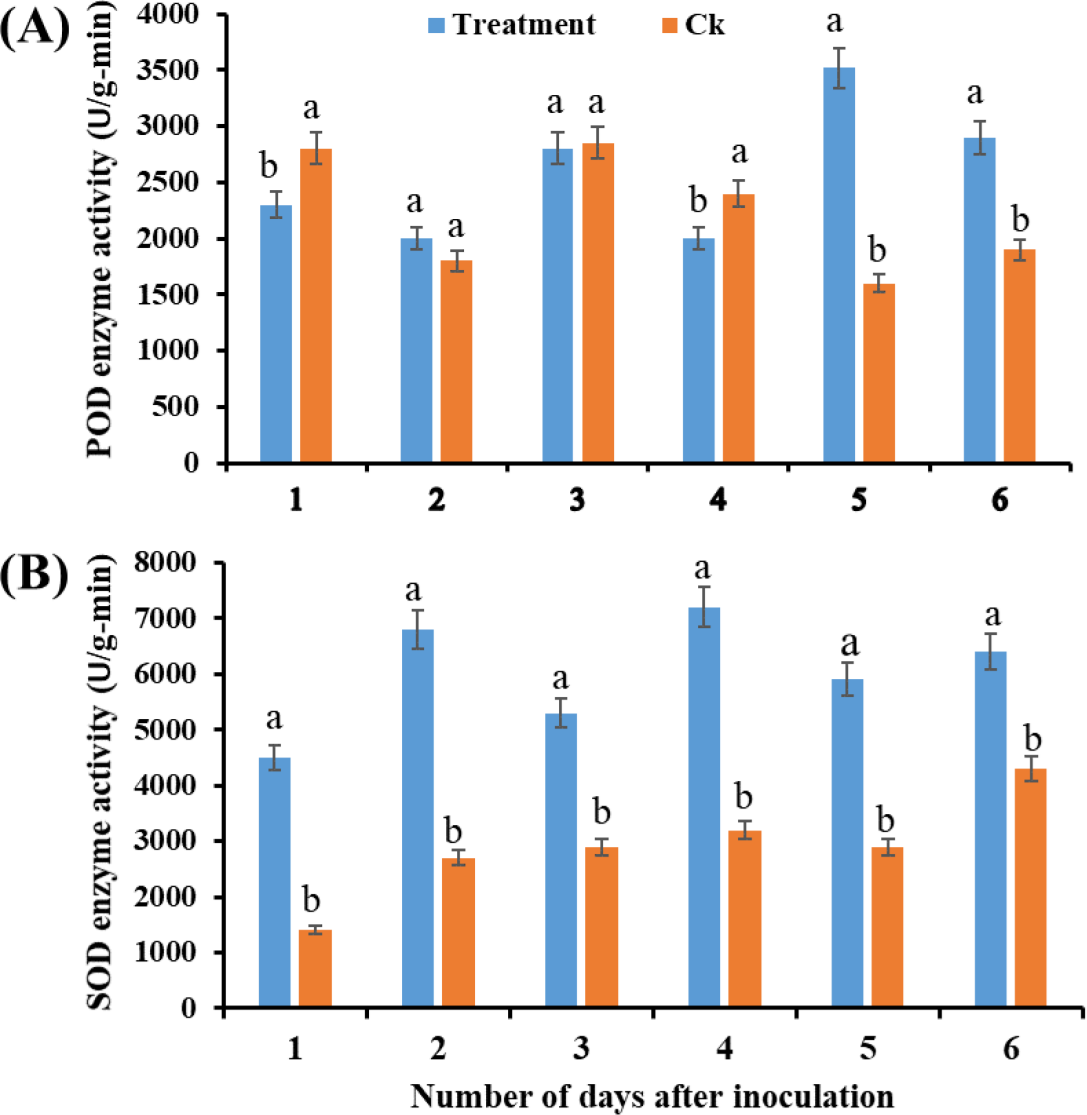
Effect of *Nigrospora oryzae* infection on antioxidant enzyme activities in sugarcane seedlings. **(A)** POD activity and **(B)** SOD activity. Values represent means ± SD (n = 3). Different letters above error bars indicate significant differences (ANOVA, *p* < 0.05). POD, peroxidase; SOD, superoxide dismutase.

**Figure 8:**
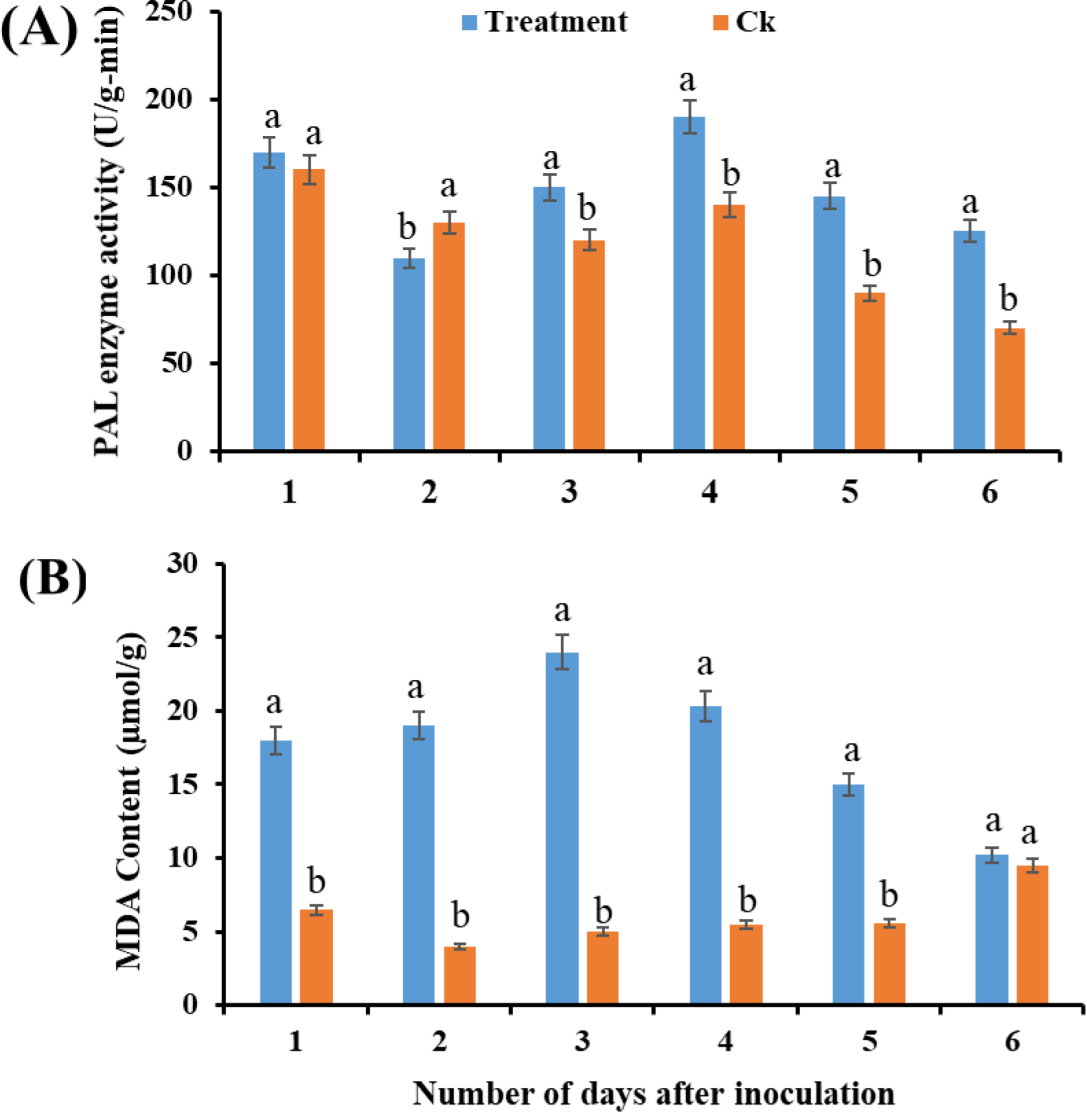
Effect of *Nigrospora oryzae* infection on antioxidant enzyme activities in sugarcane seedlings. **(A)** PAL activity and **(B)** MDA activity. Values represent means ± SD (n = 3). Different letters above error bars indicate significant differences (ANOVA, *p* < 0.05). PAL, phenylalanine ammonia-lyase; MDA, malondialdehyde.

Infection by *N. oryzae* significantly increased the activity of antioxidant enzymes in sugarcane seedlings. The activities of SOD and POD were markedly higher in infected seedlings than in controls across multiple time points (ANOVA, *p* < 0.05; **Figure 7**). Similarly, PAL activity and MDA content were significantly elevated in infected seedlings compared with controls (ANOVA, *p* < 0.05; **Figure 8**), indicating enhanced oxidative stress and activation of defense-related biochemical pathways.

### 3.8 Fungicide screening

Fungicide sensitivity assays revealed a clear hierarchy in the inhibitory activity of the eight tested compounds against *N. oryzae*. Among them, 60% pyraclostrobin + metiram was by far the most potent, achieving 95.24% inhibition at the test concentration and exhibiting the lowest EC_50_ value (0.01 μg mL^−1^). Its performance was significantly superior to all other treatments (ANOVA, *p* < 0.05; **Tables 1**, **2**), identifying it as the most effective candidate for disease management; 10% difenoconazole ranked second, providing 83.05% inhibition at 50 μg mL^−1^ with an EC_50_ of 9.698 μg mL^−1^. Although its efficacy declined more rapidly at lower concentrations compared with pyraclostrobin + metiram, it still demonstrated strong and consistent antifungal activities. A moderate level of control was achieved with 75% chlorothalonil, which produced 53.17% inhibition at the highest tested concentration and an EC_50_ of 50.149 μg mL^−1^, suggesting that only relatively high doses would be effective.

**Table 2:** EC_50_ estimates (μg mL^−1^) for fungicides against *Nigrospora oryzae* based on four-parameter log-logistic fits (LL.4) to inhibition (%) versus concentration using data from **Table 1**.

Fungicide	r	EC_50_ (μg/mL)	Interpretation
75% Chlorothalonil	0.982	**13.2**	Effective
10% Difenoconazole	0.993	**1.03**	Very potent
46% Copper hydroxide	0.611	**>10,000**	Ineffective
60% Pyraclostrobin + metiram	0.964	**0.01**	**Extremely potent (best)**
50% Carbendazim	0.958	**363**	Weak
3% Metalaxyl + hymexazol	0.903	**746**	Weak
25% Myclobutanil	0.881	**3,650**	Poor
3% Zhongshengmycin	0.841	**1,020**	Poor

Values are point estimates with 95% confidence intervals in parentheses. Lower EC_50_ indicates greater *in vitro* potency. “—” denotes not estimable (inhibition did not reach 50% within the tested range or the model failed fit criteria). Bold EC_50_ values indicate the lowest EC_50_ (most potent/‘best’ inhibition among tested fungicides).

In contrast, 25% myclobutanil showed limited activity, reaching only 39.49% inhibition at 50 μg mL^−1^ and presenting a high EC_50_ of 350.727 μg mL^−1^. The remaining fungicides—50% carbendazim, 3% metalaxyl + hymexazol, 3% zhongshengmycin, and especially 46% copper hydroxide—exhibited poor to negligible inhibition, with copper hydroxide being virtually ineffective (4.70% inhibition; EC_50_ = 8,817.992 μg mL^−1^). Collectively, these results indicate that only a subset of the tested fungicides, particularly pyraclostrobin + metiram and difenoconazole, possess strong inhibitory potential against *N. oryzae*.

## 4 Discussion

### 4.1 Pathogen biology and host–pathogen interaction

This study provides the first integrated characterization of *N. oryzae* associated with sugarcane leaf spot and clarifies key aspects of its infection biology, pathogenicity, host physiological responses, and fungicide sensitivity. Although the current incidence and economic impact of this disease under natural field conditions have not yet been quantified, observations made during sample collection indicated that leaf spot symptoms were sporadic and strongly associated with plants showing visible mechanical damage or stress. Our findings demonstrate that *N. oryzae* acts as a wound-dependent opportunistic pathogen in sugarcane, consistent with previous reports in other hosts where infection typically requires mechanical injury or weakened tissue (Liu et al., 2021; Chen, 2020). The absence of symptoms on intact leaves confirms that the pathogen cannot penetrate healthy epidermal tissue, underscoring the role of physical damage such as insect feeding, hail injury, and harvest-related wounds as critical infection courts. This wound dependency provides a plausible explanation for the irregular and localized nature of disease occurrence in the field, where infection is likely limited to areas experiencing frequent tissue injury rather than widespread epidemic development. The lesions observed on wounded leaves, characterized by necrotic spots with darkened centers, closely align with symptom descriptions for cotton rose (Wang et al., 2022), ginger (Han et al., 2021), and wild rice (Lu et al., 2023). This suggests that *N. oryzae* employs a comparable necrotrophic strategy across hosts. The rapid lesion expansion observed within several days of inoculation further supports its opportunistic lifestyle and highlights its potential threat under field conditions, where wounds are common.

Inoculated plants exhibited marked reductions in total chlorophyll, indicating impaired photosynthetic capacity. Similar chlorophyll loss has been documented in ginger and barley infected by *N. oryzae* and related necrotrophic fungi (Liu et al., 2021; Geetha et al., 2005). Elevated MDA levels in infected leaves suggest enhanced lipid peroxidation and membrane damage, which are typical markers of oxidative stress during pathogen challenge (Song et al., 2022). The significant increases in SOD and POD activities indicate the activation of the antioxidant system as the plant attempts to detoxify reactive oxygen species (ROS). Enhanced PAL activity further suggests the stimulation of the phenylpropanoid pathway, which contributes to the lignification and synthesis of antimicrobial phenolics (Wang et al., 2024). Together, these responses reflect a typical host reaction to necrotrophic pathogens, which often provoke intense oxidative bursts that contribute to cell death and lesion formation. In the context of field infections, such wound-induced oxidative responses may inadvertently facilitate pathogen colonization by accelerating localized cell death at injury sites, thereby favoring necrotrophic establishment. *N. oryzae* exhibited optimal mycelial growth at 25°C–30°C and pH 7, which is consistent with reports from other hosts and environmental isolates (Widmer et al., 2006; Liu et al., 2021). These conditions closely align with the climate of many sugarcane-growing regions, particularly in subtropical areas, suggesting that the pathogen can readily proliferate in the field when temperatures are warm and humidity is high.

### 4.2 Fungicide efficacy and integrated disease management

Fungicide screening revealed substantial variability in *N. oryzae*’s sensitivity to different active ingredients, and colony area-based inhibition showed greater differentiation among chemistries than traditional diameter measurements. The mixture of pyraclostrobin and metiram exhibited the highest inhibitory activity, achieving >90% inhibition across all concentrations and an exceptionally low EC_50_ (~0.01 μg mL^−1^). This result reflects the rapid shutdown of mitochondrial respiration by strobilurins (Fungicide Resistance Action Committee (FRAC) 11) (**Table 3**), which block electron transfer at the Qo site of Complex III, combined with the protective action of metiram. Such potency suggests that this formulation is particularly effective for the rapid suppression of early infection and sporulation, aligning with prior reports of strong quinone outside inhibitor (QoI) performance against *Nigrospora* species in other crops (Massi et al., 2021; Yin et al., 2023). Difenoconazole (FRAC 3) ranked second in efficacy (EC_50_ ≈ 1 μg mL^−1^). Its inhibition of ergosterol biosynthesis, which limits hyphal elongation and membrane formation, is consistent with its broad-spectrum activity against many ascomycetes. Given its systemic properties and reliable performance, difenoconazole is likely to be effective as both a preventive and early curative option against *N. oryzae*. Chlorothalonil showed moderate inhibitory activity (EC_50_ ≈ 13 μg mL^−1^). As a multi-site protectant (FRAC M05) that disrupts protein thiol groups, its moderate performance agrees with earlier observations that *Nigrospora* isolates often show partial tolerance to multi-site fungicides. Nonetheless, chlorothalonil may still contribute to resistance management when used in alternation with more potent chemistries. In contrast, copper hydroxide, myclobutanil, metalaxyl + hymexazol, carbendazim, and zhongshengmycin exhibited poor inhibition, yielding EC_50_ values exceeding 350–10,000 μg mL^−1^. These concentrations are far above field-applicable rates, indicating intrinsic or acquired tolerance. Several mechanistic explanations align with this observation: metalaxyl targets oomycetes rather than ascomycetes, carbendazim resistance is widespread globally, and copper-based formulations primarily target bacterial pathogens rather than filamentous fungi. Similar poor sensitivity to copper compounds has been reported in other *Nigrospora* isolates (Hawkins, 2024).

**Table 3:** FRAC code classification and mode of action (MOA).

Fungicide	FRAC code	Mode of action	Description for text
Pyraclostrobin	11	QoI inhibitor	Inhibits mitochondrial respiration at the Qo site of cytochrome *b*
Metiram	M03	Multi-site inhibitor	Disrupts multiple metabolic processes at the cell membrane
Difenoconazole	3	DMI inhibitor	Inhibits sterol (ergosterol) biosynthesis
Carbendazim	1	MBC inhibitor	Binds β-tubulin; disrupts mitosis
Copper hydroxide	M01	Multi-site copper	Denatures proteins and enzymes via copper ions
Mancozeb/mancozeb mixtures	M03	Multi-site dithiocarbamate	Reacts with thiol groups; enzyme denaturation

DMI, demethylation inhibitor; MBC, methyl benzimidazole carbamate.

Taken together, the EC_50_ values, inhibition profiles, and FRAC mode-of-action classifications clearly identify pyraclostrobin + metiram and difenoconazole as the only highly effective fungicidal options for *N. oryzae*. However, because QoI resistance evolves rapidly in many plant pathogenic fungi, they should not be used as single-dependency chemistries. Instead, integrated disease management should combine targeted fungicide selection with cultural practices that reduce wounding, including minimizing mechanical injury, managing insect pests that create entry points, and avoiding unnecessary handling damage. Given the wound-inducible nature of *N. oryzae*, disease development under field conditions is likely contingent upon the frequency and severity of physical damage rather than aggressive host penetration, emphasizing the importance of injury prevention as a primary disease control strategy. As *N. oryzae* infection is wound-dependent, reducing physical injury may significantly limit disease onset. When combined with rational fungicide use and rotation across FRAC groups, these strategies provide a robust approach for managing *N. oryzae* leaf spot.

## 5 Conclusion

Our study confirms *N. oryzae* as a wound-dependent pathogen of sugarcane and reveals its capacity to cause significant physiological and biochemical disruptions. The identification of effective fungicides provides immediate practical value, while insights into host–pathogen interactions lay a foundation for future breeding and integrated disease-management strategies. Field trials and population-level monitoring of *N. oryzae* will be critical steps to validate these findings under commercial production environments.

## Data Availability

The raw data supporting the conclusions of this article will be made available by the authors, without undue reservation.
